# Childhood-Onset Huntington’s Disease-Like Presentation of SCA17 with Intermediate Repeats, A Case Report

**DOI:** 10.1007/s12311-026-01979-3

**Published:** 2026-03-17

**Authors:** Meaghan Berns, Kelsey Jensen, Laura Speltz, Leonardo Brito Almeida

**Affiliations:** 1 Department of Neurology, University of Minnesota, Minneapolis, MN USA; 2 Department of Neurology, Allina Health, Minneapolis, MN USA; 3 Department of Neurology, Norman Fixel Institute for Neurological Diseases, University of Florida, Gainesville, FL USA

**Keywords:** Ataxia, Chorea, SCA17, HDL-4, Movement Disorder

## Abstract

Objectives: In Spinocerebellar Ataxia type 17 (SCA17), penetrance is determined by repeat number of the CAG/CAA trinucleotide in the TATA-binding protein (TBP) gene. The intermediate range (41–48) exhibits only partial penetrance but may manifest a Huntington’s disease-like (HDL-4) syndrome with onset at age 50–60 years. We report a unique case of HDL-4 with childhood onset despite intermediate range repeats. Methods: This patient was evaluated at the University of Minnesota Movement Disorders Clinic. Standard Protocol Approvals, Registrations, and Patient Consents: Informed consent to disclose was obtained from the legal guardian of the subject of this report. Results: A 14-year-old male of Chinese descent presented with generalized dystonic and choreoathetoid movements since age 11 alongside motor delays, epilepsy, and speech, balance, and coordination dysfunction since infancy. Family history was limited given his adoptive status. Subsequent workup confirmed SCA17 with repeat numbers of 43 and 37 in the intermediate range. Discussion: This is the first case of intermediate range SCA17 of childhood-onset, emphasizing the evolving classification of intermediate vs. full penetrance while suggesting that the TBP repeat length may not be the sole determinant of disease onset and severity. It also highlights the importance of repeat expansion panels when investigating complex movement disorders.

## Introduction

Spinocerebellar ataxia type 17 (SCA17) is a rare, autosomal dominant disorder caused by a CAG/CAA repeat expansion in the *TATA-binding protein (TBP)* gene [1]. The phenotype is highly variable and can include ataxia, chorea, dementia, parkinsonism, seizures, and psychiatric symptoms [1]. Unaffected individuals have ≤ 40 CAG/CAA repeats, while those with ≥ 49 repeats have full disease penetrance [1]. There is an intermediate range of 41–48 repeats with an associated partial penetrance of 50%, and a Huntington’s disease-like (HDL-4) syndrome in this group is reported with typical onset at 50–60 years of age [1, 2].

## Case Report

We report a unique case of childhood-onset HDL-4 in a 14-year-old boy of Chinese descent with generalized hyperkinetic movements first noted at age 4, progressing to become more prominent and bothersome by age 11. Family history is limited given his adoptive status as an infant. He had a history of motor and some cognitive delays, hypotonia, severe epilepsy that only achieved clinical control following initiation of levetiracetam at age 18 months, as well as both dysarthria and truncal ataxia since he was a toddler. He also experienced moderate-severe bilateral congenital sensorineural hearing loss secondary to a *Gap Junction beta-2 (GJB2)* mutation, thought to cause a non-syndromic hearing loss without other known neurologic manifestations [3]. There was no history of chronic environmental exposures, smoking, alcohol, or drugs. There were no known chronic exposures to dopamine-blocking agents.

His routine medications included levetiracetam for seizures and fluoxetine for anxiety, both being added subsequent to the onset of his hyperkinetic disorder. He also took melatonin for sleep and had diazepam and midazolam as needed for prolonged breakthrough seizures.

Serial neurological examinations at the movement disorders clinic (at the age of 14) evidenced normal to slightly reduced tone and 5/5 strength throughout. Cranial nerve exam – save for known sensorineural hearing loss – was normal, with symmetric facial movement and intact sensation. Sensation was preserved to all modalities in all extremities. Reflexes were 1 + with down-going toes bilaterally. Coordination testing showed no clear dysmetria or dysdiadochokinesia, with observation of florid hyperkinetic movements that could have been intruding into his volitional motor tasks, potentially masking underlying appendicular cerebellar findings. His movement disorder phenomenology consisted of hyperkinetic movements comprising generalized dystonia and choreoathetosis.

Workup included an unremarkable brain MRI in 2012 which was repeated in 2021, then showing left-greater-than-right mesial temporal sclerosis, but no signs of cerebellar atrophy. His most recent EEG in 2019 demonstrated no epileptiform activity. Laboratory findings including blood counts, complete metabolic panel, thyroid hormone, parathyroid hormone, streptolysin O and DNase B antibody, and urine purine/pyrimidine levels were also unrevealing. Initial genetic studies included a normal chromosomal microarray and methylation study for Prader-Willi as well as sequencing of the *GJB2* gene which returned positive for homozygous mutations, explaining his hearing loss only. Whole exome sequencing (WES) identified two heterozygous variants of uncertain significance (VUS) in the dihydropyrimidine dehydrogenase (DPYD) gene, which is autosomal recessive and deemed to not be related with his presentation. Huntington’s disease testing was negative as was mitochondrial genome analysis. Subsequent genetic workup with The University of Chicago Genetic Services Laboratory Ataxia Repeat Expansion Panel (genes TBP, ATXN1, ATXN2, ATXN3, CACNA1A, ATXN7, ATXN8OS/ATXN8, ATXN10, PPP2R2B, FGF14, ATN1, FXN, FMR1, and RFC1) showed repeat numbers of 43 and 37 in the *TBP* gene. The genetic results were consistent with spinocerebellar ataxia (SCA)17 (HDL-4) with the number of repeats falling within the intermediate range (41–48 repeats). No pathogenic variants in the *STUB1* gene - previously reported to precipitate disease expression in the intermediate range [4] - were found.

Standard Protocol Approvals, Registrations, and Patient Consents: Informed consent to disclose was obtained from the legal guardian of the subject of this report.

## Discussion

This case exemplifies the phenotypic variability of SCA17, with special attention to an early onset with an intermediate number of repeats. While childhood-onset SCA17 has been reported, these are typically associated with full penetrance due to a high number of repeats, consistent with genetic disorders associated with nucleotide repeats [1, 2]. The typical onset of HDL-4 with an intermediate-range repeat expansion is between 50 and 60 years of age [2]. To our knowledge, this is the first case with an intermediate-range repeat expansion of childhood-onset.

In regards to his clinical presentation, it remains unknown whether the cognitive delay would be part of his SCA17 presentation. While developmental delay is not a typical feature of SCA17, it is important to note that the majority of cases with intermediate repeats occur much later in life (50s to 60s). Therefore, the association with cognitive involvement/delay remains to be established for childhood-onset cases. Further, the patient had a history of uncontrolled epilepsy (which can be a feature of SCA17) in his infancy, with evidence of mesial temporal sclerosis on imaging, raising concerns that a component of his cognitive involvement may have been secondary to uncontrolled epilepsy. Lastly, one may note the lack of cerebellar atrophy (which is found in SCA17) on brain imaging. The authors hypothesize that, given his young age, while he historically had ataxic symptoms prior to the evolution of hyperkinetic movements to a disabling level, it is conceivable that his underlying ataxia remains insufficient to produce evidence of cerebellar atrophy/degeneration on imaging.

We believe that this unique presentation adds to the growing literature and phenotypic range of SCA17. It also emphasizes that the classification of intermediate vs. full-penetrance in this condition is evolving, particularly due to the rarity of SCA17 and its variable presentations. Our case hypothesizes that the *TBP* repeat length may not be the sole determinant of disease onset and severity and that other genetic or environmental factors may contribute to early disease expression which are yet to be fully explored, similar to the role of concomitant *STUB1* mutations. Nonetheless this example highlights the importance of including repeat expansion panels in the diagnostic workup for patients with complex movement disorders when initial workup is inconclusive, and should raise awareness for the variety of phenotypical presentations of these newly found mutations.

## Data Availability

The data that support the findings of this study are not openly available due to reasons of sensitivity and are available from the corresponding author upon reasonable request.
